# The use of DNA barcodes to estimate phylogenetic diversity in forest communities of southern China

**DOI:** 10.1002/ece3.5128

**Published:** 2019-04-03

**Authors:** Jiajia Liu, Juan Liu, You-Xia Shan, Xue‐Jun Ge, Kevin S. Burgess

**Affiliations:** ^1^ Key Laboratory of Plant Resources Conservation and Sustainable Utilization, South China Botanical Garden The Chinese Academy of Sciences Guangzhou China; ^2^ Collaborative Innovation Center of Jiangxi Typical Trees Cultivation and Utilization Jiangxi Agriculture University Nanchang China; ^3^ Department of Biology, College of Letters and Sciences Columbus State University, University System of Georgia Columbus Georgia

**Keywords:** Bayesian tree, ITS, *matK*, phylogenetic inference, *rbcL*, subtropical forest

## Abstract

To elucidate potential ecological and evolutionary processes associated with the assembly of plant communities, there is now widespread use of estimates of phylogenetic diversity that are based on a variety of DNA barcode regions and phylogenetic construction methods. However, relatively few studies consider how estimates of phylogenetic diversity may be influenced by single DNA barcodes incorporated into a sequence matrix (conservative regions vs. hypervariable regions) and the use of a backbone family‐level phylogeny. Here, we use general linear mixed‐effects models to examine the influence of different combinations of core DNA barcodes (*rbcL*, *matK*, ITS, and ITS2) and phylogeny construction methods on a series of estimates of community phylogenetic diversity for two subtropical forest plots in Guangdong, southern China. We ask: (a) What are the relative influences of single DNA barcodes on estimates phylogenetic diversity metrics? and (b) What is the effect of using a backbone family‐level phylogeny to estimate topology‐based phylogenetic diversity metrics? The combination of more than one barcode (i.e., *rbcL* + *matK *+ ITS) and the use of a backbone family‐level phylogeny provided the most parsimonious explanation of variation in estimates of phylogenetic diversity. The use of a backbone family‐level phylogeny showed a stronger effect on phylogenetic diversity metrics that are based on tree topology compared to those that are based on branch lengths. In addition, the variation in the estimates of phylogenetic diversity that was explained by the top‐rank models ranged from 0.1% to 31% and was dependent on the type of phylogenetic community structure metric. Our study underscores the importance of incorporating a multilocus DNA barcode and the use of a backbone family‐level phylogeny to infer phylogenetic diversity, where the type of DNA barcode employed and the phylogenetic construction method used can serve as a significant source of variation in estimates of phylogenetic community structure.

## 1 INTRODUCTION

Plant DNA barcodes, based on either single or multilocus regions of the chloroplast and/or nuclear genomes, have been applied to questions in community ecology (Kress et al., 2009; Valentini, Pompanon, & Taberlet, 2009). Estimates of phylogenetic genetic diversity can be used to quantify the evolutionary and ecological processes associated with community assembly, composition, and structure at different spatiotemporal scales (Cavender‐Bares, Kozak, Fine, & Kembel, 2009; Helmus, Bland, Williams, & Ives, 2007; Mouquet et al., 2012; Webb, 2000). The branch lengths and topology of community phylogenies can influence estimates of phylogenetic diversity in different ways (Boyle & Adamowicz, 2015; Mazel et al., 2016; Swenson, 2009). For estimates based on DNA barcodes, the metric used to assess phylogenetic diversity may be influenced by the evolutionary rate of the barcode(s) employed. For example, DNA barcode regions that are phylogenetically conservative or hypervariable may under‐ or overestimate phylogenetic diversity, respectively. The effect of barcode region (and their combinations) on estimates of phylogenetic diversity metrics has not been empirically tested and may be a potential source of variation that requires consideration when assessing community phylogenetic structure.

Estimates of phylogenetic diversity may be also influenced by the type of phylogenetic construction method employed. Typically, tree topologies at deep phylogenetic nodes (e.g., family level) that have been inferred with a limited set of barcodes are largely incongruent with broadly accepted patterns of taxonomic relationships (e.g., APG IV; Byng et al., 2016). To constrain deep phylogenetic nodes and follow broadly accepted phylogenetic patterns, supertree methods (Bininda‐Emonds & Sanderson, 2001; Webb & Donoghue, 2005) can be combined with DNA barcode sequence data (Erickson et al., 2014; Kress et al., 2010) to provide more accurate depictions of topology. Furthermore, the incorporation of a backbone phylogeny can provide more accurate estimates of the branch lengths (Boyle & Adamowicz, 2015) and potentially affect the metrics of phylogenetic community diversity (Swenson, 2009). Since ecological and evolutionary processes might operate at different phylogenetic depths (Mazel et al., 2016), it seems reasonable that phylogenetic diversity metrics that are sensitive to processes operating at deep phylogenetic depths may be strongly influenced by combining supertree methods with DNA barcode sequence data, whereas those estimates that are largely capturing diversity at the tips of the phylogeny may be less influenced, although this remains to be tested.

In contrast to the branch length‐based metrics, several phylogenetic diversity metrics (e.g., PAE, the relationship between species evolutionary distinctiveness and abundance; IAC, the imbalance of abundances at higher clades) have been developed to capture information on both the topology and branch lengths of phylogenies connecting the species of a community (Cadotte et al., 2010; Krajewski, 1994; Vanewright, Humphries, & Williams, 1991). These topology‐based metrics have also been shown to be valuable for predicting patterns of abundance, community composition, and ecosystem functioning (Cadotte et al., 2010; Liu et al., 2018; Liu, Zhang, et al., 2015), but are seldomly evaluated in terms of how the branch lengths and topologies of community phylogenies may affect estimates of community phylogenetic diversity. Here, we predict that the use of a backbone phylogeny will have a strong influence on topology‐based metrics.

To assess the potential variance in estimates of phylogenetic diversity associated with DNA barcodes and phylogeny construction methods, we first constructed a series of phylogenies, using Bayesian tree inference, for two distinct tropical forest communities that vary in elevation in the Dinghushan National Nature Reserve, Guangzhou, China. Specifically, we sampled two plastid gene regions (*rbcL* + *matK*) and the nuclear ribosomal internal transcribed spacers (ITS and ITS2 as part of the ITS region but with considerable power in species identification and resolution, see Chen et al., 2010) for all trees in each plot and constructed a series of phylogenies using different barcode combinations. To investigate the effects of supertree methods on estimates of phylogenetic diversity, we constructed another series of phylogenies with backbone family‐level phylogenies based on APG IV (Byng et al., 2016). Taking a multi‐model comparative approach, we assessed the relative contribution of single and multilocus barcodes, family‐level backbone, and their combinations to predict the variance in estimates of phylogenetic diversity metrics. We address the following questions: (a) What are the relative influences of single DNA barcodes on estimates on phylogenetic diversity metrics? (b) What is the effect of using a backbone family‐level phylogeny to estimate phylogenetic diversity metrics?

## 2 MATERIALS AND METHODS

### 2.1 Study sites

**Figure 1:**
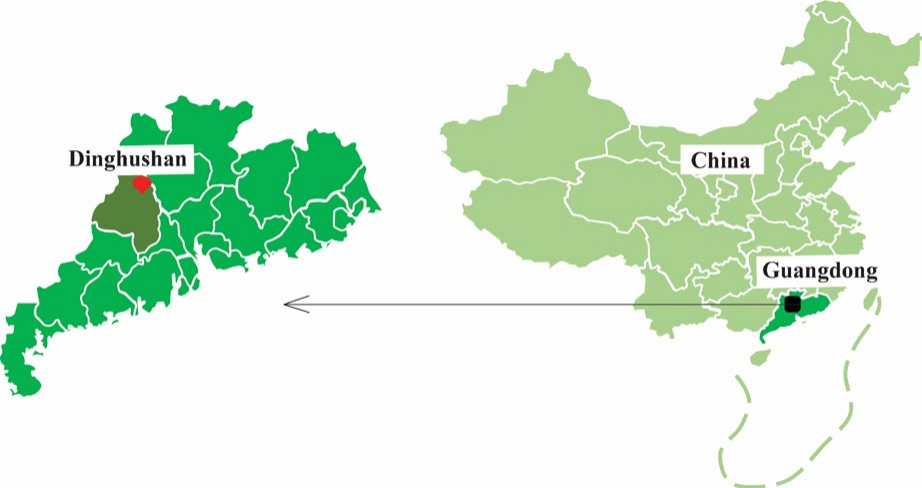
Map of the study sites on Dinghu Mountain, Guangzhou, Guangdong Province, China

Both study plots were located at the Dinghushan National Nature Reserve, Guangdong province, South China (Figure 1: 23°10′N, 112°31′E; 23°10′N, 112°32′E), where the mean annual temperature is 21.0°C (range: −0.2°C to 38.1°C) and mean annual rainfall is 1,927 mm (Liu, Yan, et al., 2015). One plot is located in a subtropical mountain evergreen forest (600 m a.s.l.), while the other plot is located in a subtropical valley rain forest (100 m a.s.l.). Both plots have the same sampling area (1 ha) and similar arboreal species richness. There were a total of 114 trees with the abundance of each species being calculated by counting the number of individuals at breast height >10 cm in both plots. The mountain evergreen forest plot had 75 species with 41 unique species, and the valley rain forest plot had 73 species with 39 unique species. We list the detailed species information in Supporting Information Table S1.

### 2.2 Community phylogenies

An exhaustive description of the methods for DNA extraction, PCR amplification, and sequencing can be found in Liu, Yan, et al. (2015). Here, we briefly describe the methods for phylogenetic construction. For the 114 species across both plots, we aligned *rbcL* and *matK* using MAFFT (Katoh & Standley, 2013) and then eliminated divergent regions using Gblocks (Castresana, 2000). We aligned ITS and ITS2 using SATé (Liu et al., 2012). We then concatenated subsets of the *rbcL*, *matK*, ITS, and ITS2 sequences to generate a total of seven super matrices: (a) *rbcL* + *matK*, (b) *rbcL* + ITS, (c) *rbcL* + ITS2, (d) *matK* + ITS, (e) *matK* + ITS2, (f) *rbcL* + *matK* + ITS, (g) *rbcL* + *matK* + ITS2.

To assess the influence of a constrained family‐level backbone on community phylogenetic diversity metrics, we constructed a total of fourteen species‐level phylogenies based on the seven super matrices: one set based on Bayesian phylogenies and a second set based on Bayesian phylogenies with a constrained backbone topology at the family level based on the APG IV system (Byng et al., 2016). We then selected the best model of nucleotide substitution based on the lowest Akaike information's criterion (AIC) for each barcode region using the function “modelTest” in the *phangorn* library (Schliep, 2011) in R (R Core Team, 2016). For all barcode combinations, modelTest found that the best model was the generalized time reversible (GTR) model with a gamma distribution parameter describing among‐site rate variation and a proportion of invariant sites parameter. We constructed all Bayesian phylogenies in MrBayes 3.2.5 (Ronquist et al., 2012) using four chains with 1,000,000 generations, a sampling and diagnostic frequency of 100, and a 20% burn in. We chose one representative of an early diverging gymnosperm lineage, *Cunninghamia lanceolata*, as the root for the Bayesian phylogenies. We then used a semi‐parametric rate‐smoothing method to transform the phylogeny to an ultrametric tree using the “chronopl” function with *λ* value 1,000 in the R *ape* library (Paradis, Claude, & Strimmer, 2004). For Bayesian phylogenies without family‐level backbone constraint, we ranked all the post topologies by the symmetric distance with the backbone topology at the family level based on APG IV system using the function “treedist” in the R *phangorn* library (Schliep, 2011). Then we selected the top ranking 500 topologies for further analysis. We also randomly selected 500 topologies for Bayesian phylogenies with backbone for comparison. We used these selected topologies to estimate the posterior probabilities of the nodes for the Bayesian phylogenies and the Bayesian phylogenies with backbone, respectively (Figure S1–S7).

### 2.3 Phylogenetic diversity metrics

For each of the fourteen Bayesian phylogenies (7 supermatricies with and 7 supermatricies without the backbone), we calculated several measures of phylogenetic diversity for all plants in the data set as well as at the plot level: Faith's PD, which sums all phylogenetic branch lengths (Faith, 1992); mean pairwise distance (MPD), which is the average distance separating all pairs of species of a community on the phylogenetic tree (Webb, Ackerly, McPeek, & Donoghue, 2002); and mean nearest taxon distance (MNTD), which is the average of the shortest phylogenetic distance for each species to its closest relative in the assemblage (Webb et al., 2002). We calculated MPD and MNTD using a species presence/absence matrix as well as a species abundance matrix. We denoted MPD_ed_, MNND_ed _for the abundance‐weighted versions of the metrics, respectively. In addition, we calculated (a) a metric of phylogenetic‐abundance evenness (PAE), which evaluates the relationship between the abundance and the distribution of terminal branch lengths (Cadotte et al., 2010) and (b) the imbalance of abundances at higher clades (IAC), which encapsulates the distribution of individuals across the nodes in the phylogeny (Cadotte et al., 2010). These diversity measures were chosen because of their wide use in ecology and conservation and because they represent measures of diversity that are based upon either branch lengths or tree topology.

### 2.4 Linear mixed‐effects models

To determine the effects of single and multilocus DNA barcodes, family‐level backbone, and their combinations on each measure of phylogenetic diversity, we constructed a series of linear mixed‐effects models using the “lme” function in the *nlme* library in R (Jose Pinheiro, Bates, DebRoy, Sarkar, & Team, 2016). The general form of the GLMM is as follows:

$$\text{log}\left(\text{phylogenetic}\;\text{diversity}\right)=\alpha +\delta_{\text{plot}}+rbcL\beta_{1}+matK\beta_{2}+\left(\text{ITS}/\text{ITS2}\right)\beta_{3}+\text{backbone}\beta_{4}.$$

where *rbcL*, *matK*, ITS, ITS2, and the use of a family‐level backbone phylogeny were set as fixed effects (not including the global intercept, *α*), and the plots (100 and 600 m) are random effects. We modeled the plots as random intercepts (*δ*_plot_) to account for plot‐level differences in measures of phylogenetic diversity that were unrelated to the particular barcodes and the use of a family‐level backbone phylogeny. To meet the assumptions of normality, we log‐transformed all measures of phylogenetic diversity. We evaluated model support using Akaike's Information Criterion corrected for small sample sizes (AIC_c_; Burnham & Anderson, 2002,2004). To describe the proportion of variance explained by just the fixed factors and by the fixed and random factors together, we used the function “r.squaredGLMM” in the library *MuMIn* in R to calculate marginal *R* ^2^ and conditional *R* ^2^, respectively (Barton, 2018; Nakagawa & Schielzeth, 2013). To check the robustness of multi‐model inferences according to random sampling, we randomly resampled the suites of estimates of phylogenetic diversity 100 times and reran the multi‐model inference for random datasets each time. The model ranks were consistent among random samples. Here, we only present the multi‐model inference results based on original measures of phylogenetic diversity. To provide a relative rank of the importance of main predictors, we calculated standardized coefficients (*β*_n_/SE_n_) for each *n *term in the models featured in each subset, averaged these across all models based on AIC*_c_* weights (*w*AIC*_c_*) (re‐calculating Σ*w*AIC*_c_* = 1 over the models in which each term appeared), and then calculated the mean and confidence intervals (95%) of standardized coefficients for each term.

## 3 RESULTS

Of the 24 general linear mixed‐effect models that were constructed (including the intercept‐only model), estimates of phylogenetic diversity based on models that included multi‐locus barcodes had higher rankings than those based on single barcodes (Table 1, Supporting Information Table S2–S8). The top‐rank models for all metrics except IAC included *rbcL*, *matK*, ITS, and family‐level backbone (*w*AIC*_c_* = 0.999 for PD, MPD, MPD_ed_, MNTD, MNTD_ed_, and PAE) (Table 1 and Supporting Information Tables S2–S7), which accounted for >9% of the variances explained for each estimate. By contrast, ITS2 instead of ITS was included in the most parsimonious models for IAC (*w*AIC*_c_* = 0.999; Table 1 and Supporting Information Table S8). However, IAC estimates were much less dependent on the combination of DNA sequence data and phylogeny construction method compared to estimates for other metrics ($R_{m}^{2}$ < 0.1%; Table 1 and Supporting Information Table S8).

**Table 1:** General linear mixed‐effect model (GLMM) results for phylogenetic diversity metrics as a function of several fixed factors and hierarchical random factors

Metric	Model	AIC_c_	ΔAIC_c_	*w*AIC_c_	$R_{m}^{2}$	$R_{c}^{2}$
PD	~B + M + R + I	−21,519	0	0.999	9.92	72.98
	~M + R + I	−21,483	36	<0.001	9.84	72.90
	~B + M + I	−20,854	665	<0.001	8.61	71.62
MPD	~B + M + R + I	−20,165	0	0.999	31.34	32.18
	~M + R + I	−20,105	60	<0.001	31.04	31.88
	~B + M + R + I2	−19,618	547	<0.001	28.65	29.47
MPD_ed_	~B + M + R + I	−20,835	0	0.999	25.03	45.34
	~M + R + I	−20,806	29	<0.001	24.91	45.22
	~B + M + R + I2	−20,103	732	<0.001	22.12	42.34
MNTD	~B + M + R + I	−6,867	0	0.999	11.88	78.80
	~M + R + I	−6,848	20	<0.001	11.85	78.76
	~B + M + I	−4,935	1933	<0.001	9.09	74.69
MNTD_ed_	~B + M + R + I	−11,181	0	0.999	15.11	63.78
	~M + R + I	−11,150	31	<0.001	15.03	63.69
	~B + M + R + I2	−10,583	598	<0.001	13.52	62.25
PAE	~B + M + R + I	−35,388	0	0.999	21.14	46.87
	~B + R + I	−34,775	613	<0.001	18.75	44.48
	~M + R + I	−34,729	660	<0.001	18.57	44.29
IAC	~B + M + R + I2	−53,553	0	0.999	0.034	99.893
	~B + M + R + I	−53,460	94	<0.001	0.033	99.892
	~B + M + R	−53,434	120	<0.001	0.033	99.892

Notes

Fixed factors are single plant barcodes (M = *matK*, R = *rbcL*, I = ITS, I2 = ITS2) and family‐level backbone (B). Random factors are plots (100 and 600 m). Metrics are shown for seven phylogenetic diversity metrics (PD: phylogenetic diversity, MPD: mean pairwise distance, MPD_ed_: abundance‐weighted MPD, MNTD: mean nearest taxon distance, MNTD_ed_: abundance‐weighted MNTD, PAE: phylogenetic‐abundance evenness, IAC: imbalance of abundance among clades). Values are shown for the information‐theoretic Akaike's information criterion corrected for small samples (AIC_c_), change in AIC_c_ relative to the top‐ranked model (ΔAIC_c_), AIC_c_ weight (*w*AIC_c_, model probability), and the marginal and total variance explained ($R_{m}^{2}$, $R_{c}^{2}$) as a measure of the model's goodness‐of‐fit. The top 3 models are listed; the full table is shown in Supporting Information Table S2–S8.

**Figure 2:**
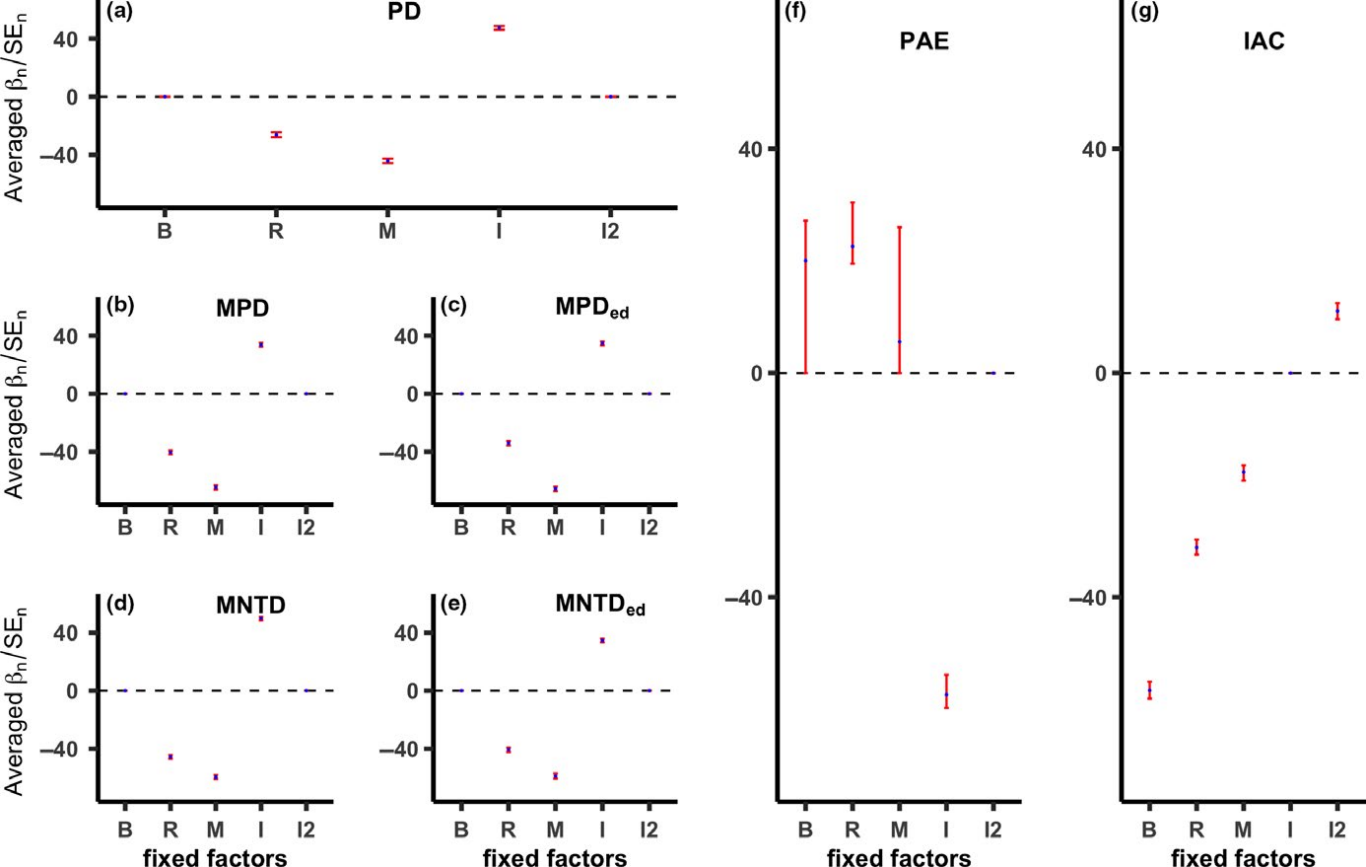
Averaged model standardized coefficients for each term considered in the general linear mixed‐effect model sets to model the variances in measures of phylogenetic diversity of subtropical forest communities in China. Negative values indicate a negative relationship to estimates of phylogenetic diversity. *β*_n_ = estimated model term (*n*) coefficient, SE_n_ = term standard error, B = family‐level backbone, R = *rbcL*, M = *matK*, I = ITS, I2 = ITS2. Analyses include a series of estimates of phylogenetic diversity (PD = phylogenetic diversity, MPD = mean pairwise distance, MPD_ed_ = abundance‐weighted MPD, MNTD = mean nearest taxon distance, MNTD_ed_ = abundance‐weighted MNTD, PAE = phylogenetic‐abundance evenness, IAC = imbalance of abundance among clades). Shown in blue points and red error bars are the mean and confidence interval (95%) of the bootstrapping values of averaged model standardized coefficients for each term and each metric

For phylogenetic diversity metrics based on branch length, *rbcL*, *matK*,and ITS showed stronger effects on estimates of phylogenetic diversity than ITS2 and the backbone family‐level phylogeny (Table 1, Figure 2a–e); *matK* had the strongest effect across the branch length‐based metrics (Figure 2a–e). Family‐level backbone had profound effects on measures of phylogenetic diversity that were based on tree topology (Figure 2f,g), but the direction of its effect depended on the topology‐based metric. ITS was the most influential factor for estimates of PAE (Figure 2f), whereas family‐level backbone was the most influential factor for IAC (Figure 2g).

## 4 DISCUSSION

Our results reveal that multilocus barcodes outperform single‐locus barcodes in explaining maximum variation in estimates of phylogenetic diversity regardless of the phylogenetic reconstruction methods used, both in terms of model ranking and model‐averaged, standardized effects. This result is in line with previous meta‐analytical and experimental evidence that suggests a combination of more than one DNA barcode locus, including a phylogenetically conservative coding locus and one or more rapidly evolving barcode regions, are essential for inferring robust phylogenetic relationships among plants (Burgess et al., 2011; Fazekas et al., 2008; Hollingsworth, Forrest, et al., 2009; Kress & Erickson, 2007; Kress et al., 2009; Li et al., 2011; Liu, Yan, et al., 2015). Here, the reason for the complementary influence of DNA barcodes with different rates of evolution might be due to different ecological and evolutionary processes operating at different evolutionary time scales, which contribute differently to plant community phylogenetic structure (Mazel et al., 2016). For example, conserved DNA barcodes might provide important insight into the processes acting at long evolutionary time scales, whereas rapidly evolving barcodes might signal more recent speciation events (Webster, Payne, & Pagel, 2003).

Of the universal barcodes that were used in our models, the effects of both chloroplast DNA regions (*rbcL* & *matK*) were evident across all phylogenetic diversity metrics. Notably, *matK* tended to be a more important factor for inferring phylogenetic diversity metrics that are based on branch length methods, while *rbcL* had a greater influence on topology‐based metrics. This result suggests that *rbcL* and *matK* might be useful for estimates phylogenetic diversity for subtropical plant communities in China by establishing deep phylogenetic branches and terminal branches, respectively. Indeed, there is increasing evidence that *matK* is the most variable coding region of the angiosperm plastome and as such, in most genera, *matK* has higher species discriminatory power compared to *rbcL *(Hilu et al., 2003; Hollingsworth, Clark, et al., 2009; Liu, Yan, et al., 2015). However, our results also suggest that the influences of DNA barcodes on measures of phylogenetic diversity might be inconsistent with their discriminatory success and more dependent on the methods and metrics used, both sources of variation that will likely have broad implications for future studies.

In this study, ITS had a stronger effect than ITS2 on estimates of community phylogenetic diversity except for IAC. This result is not surprising given that ITS2 is only one of three partitions in the ITS gene region (ITS1, 5.8S, ITS2; Coleman, 2003). Collectively, the effects of ITS on estimates of phylogenetic diversity that were based on branch length methods were comparable to those of regions of the plastid genome (i.e., *matK*) using model‐averaged, standardized coefficients. Although this finding implies that ITS does not estimate branch lengths better than *matK*, ITS does show a much stronger influence on estimates of PAE than barcode regions of the plastid genome. Because PAE stresses the phylogenetic‐abundance distributions among terminal branches (Cadotte et al., 2010), our results indicate that ITS may be a better estimator, over the other three DNA barcodes, of phylogenetic relationships at the tips of the community phylogeny. Of the four DNA barcode markers used in this study, ITS has been shown to have the highest species discriminatory power due to its ability to differentiate closely related, congeneric species (Li et al., 2011). Meanwhile, ITS2 outperformed ITS in predicting the variation in IAC, which stresses the topology at deep nodes of the phylogeny. This result suggests that ITS2 may be better tool for estimating phylogenetic relationships at deep clades (Yao et al., 2010).

We found that models that included a backbone family‐level phylogeny had the highest support but only showed a strong effect for measures of phylogenetic diversity that are based on the topology of the community phylogeny. In our study system, the use of a backbone family‐level phylogeny was required to explain maximum variation in topology‐based metrics, which is consistent with our expectation that a limited number of DNA barcodes might generate inconsistent relationships deep within the phylogeny compared to broadly accepted patterns (Erickson et al., 2014). Such inconsistency might have a significant influence on measures of phylogenetic diversity, which are more sensitive to the basal topology of phylogenies. Indeed, we found evidence that the use of a backbone family‐level phylogeny was more effective on estimates of IAC than that of PAE, given PAE measures the phylogenetic‐abundance distribution among terminal branches and IAC quantifies the imbalance of abundances at deeper clades (Cadotte et al., 2010). However, the effects of enforcing a backbone for deeper relationships in the phylogeny were negligible for estimates of branch length‐based metrics.

Although the optimal combination of DNA barcodes (e.g., *rbcL* + *matK* + ITS) and the use of a backbone family‐level phylogeny served as a consistent and accurate predictor for the metrics of phylogenetic diversity considered here, the explanatory power of the top‐rank models varied depending on how phylogenetic diversity was measured. For example, mean pairwise distance (MPD) attained the highest proportion (>31%) of the explained variation, whereas the imbalance of abundances at higher clades (IAC) attained the lowest proportion (0.1%). It is generally agreed that different ecological processes (i.e., environmental filtering and limiting similarity) and evolutionary processes (i.e., local adaptation, speciation, extinction) operating at different spatiotemporal scales can contribute to community structure (Cavender‐Bares et al., 2009; Swenson, 2011). However, determining the relative contribution of ecological versus evolutionary processes contributing to community patterns can be difficult (Cavender‐Bares et al., 2009). Among the metrics of phylogenetic diversity considered, MPD was “best” explained by the combination of DNA barcodes, which is consistent with previous studies. For example, Mazel et al. (2016) showed that MPD is more sensitive to long‐term evolutionary processes compared to PD and MNTD. However, our results for IAC, which is independent of the DNA barcodes and phylogeny construction methods, suggest that ecological processes are mainly generating community phylogenetic patterns at our sites. Given that combinations of DNA barcodes, the use of a backbone family‐level phylogeny, and the plots together accounted for the substantial proportions ($R_{c}^{2}$ > 32%) of variation in a series of estimates of phylogenetic diversity, this result is in line with the view that both ecological and evolutionary processes are influencing biodiversity at our sites. Future studies should consider the inclusion of functional, environmental, and demographic data to further elucidate the underlining ecological and evolutionary mechanisms contributing to community structure.

This study examined the influence of different combinations of four core DNA barcodes and community phylogeny reconstruction methods on a series of estimates of community phylogenetic diversity metrics for two subtropical forest plots in Guangdong, southern China. There are, however, a number of additional DNA fragments including coding regions (i.e., *rpoB* & *rpoC1*) and noncoding spacer regions (i.e., *atpF‐atpH*, *trnH‐psbA*, and *psbK‐psbI*) that have been proposed as candidates for universal plant DNA barcodes (Hollingsworth, Forrest, et al., 2009). Furthermore, our power to detect influences of plant DNA barcodes and tree construction methods on estimates of phylogenetic diversity was limited by a small set of phylogenetic diversity metrics. Although these factors should be considered in future studies, our study provides insight into the magnitude of the influence of single barcodes, or their combination, and phylogeny reconstruction methods on community phylogenetic patterns. Notably, our study underscores the complexity of explaining community phylogenetic patterns, where future studies should evaluate the sensitivity of phylogenetic diversity metrics to the methods employed.

## CONFLICT OF INTEREST

None declared.

## AUTHOR CONTRIBUTIONS

JJL conceived the idea, JL collected the DNA data, JJL and YXS analyzed the data, JJL and KSB led the writing of the manuscript, and all authors contributed critically to the drafts and gave final approval for publication.

## Data Availability

The essential data in the data analysis of this paper will be archived in a public accessible repository upon acceptance.
