# Supplementary material for: The use of DNA barcodes to estimate phylogenetic diversity in forest communities of southern China
Source: Ecol Evol. 2019 Apr 3;9(9):5372–9. doi: 10.1002/ece3.5128 (PMC6509380; doi:10.1002/ece3.5128)

**Table S1.** Angiosperm species list and their associate genbank accession number of four barcodes (*rbcL*, *matK*, ITS and ITS2) and abundance in both subtropical valley rain forest plot (Plot1, Elevation 100 m) and subtropical mountain evergreen forest plot (Plot 2, Elevation 600 m).

| **Family** | **Species** | ***rbcL*** | ***matK*** | **ITS** | **ITS2** | **Plot1** | **Plot2** |
| --- | --- | --- | --- | --- | --- | --- | --- |
| Araliaceae | *Schefflera heptaphylla* | KP094266 | KP093352 | KP092539 | - | 20 | 2 |
| Aquifoliaceae | *Ilex asprella* | KP094153 | KP093246 | KP092544 | - | 11 | 0 |
| Aquifoliaceae | *Ilex memecylifolia* | KP094921 | KP093977 | KP092553 | - | 0 | 321 |
| Aquifoliaceae | *Ilex pubescens* | KP094364 | KP093446 | KP092555 | - | 1 | 0 |
| Aquifoliaceae | *Ilex triflora* | KP094396 | KP093476 | KP092559 | - | 45 | 1 |
| Arecaceae | *Caryota maxima* | KP094766 | KP093827 | #N/A | KP095988 | 0 | 146 |
| Adoxaceae | *Viburnum sempervirens* | KP094734 | KP093797 | KP092593 | - | 22 | 0 |
| Ericaceae | *Craibiodendron scleranthum* | KP094790 | KP093850 | #N/A | #N/A | 42 | 2 |
| Ericaceae | *Rhododendron henryi* | KP094753 | KP093814 | KP092601 | - | 1137 | 0 |
| Ericaceae | *Rhododendron mariae* | KP094253 | KP093341 | KP092607 | - | 14 | 0 |
| Ericaceae | *Rhododendron moulmainense* | KP094916 | KP093973 | KP092609 | - | 86 | 0 |
| Ericaceae | *Rhododendron simsii* | KP094652 | KP093715 | KP092611 | - | 4 | 0 |
| Pentaphylacaceae | *Eurya acuminatissima* | KP094924 | KP093980 | KP092638 | - | 27 | 0 |
| Pentaphylacaceae | *Eurya macartneyi* | KP095116 | KP094148 | KP092641 | - | 540 | 0 |
| Primulaceae | *Ardisia crenata* | KP094325 | KP093409 | KP092625 | - | 0 | 6 |
| Primulaceae | *Ardisia quinquegona* | KP094338 | KP093420 | KP092650 | - | 1 | 40 |
| Primulaceae | *Maesa japonica* | KP094958 | KP094008 | KP092664 | - | 0 | 1 |
| Primulaceae | *Maesa salicifolia* | KP094678 | KP093741 | KP092667 | - | 1 | 0 |
| Primulaceae | *Myrsine seguinii* | KP094788 | KP093848 | KP092669 | - | 556 | 1 |
| Symplocaceae | *Symplocos cochinchinensis* | KP094414 | KP093493 | KP092674 | - | 2 | 0 |
| Symplocaceae | *Symplocos lancifolia* | KP094151 | KP093244 | KP092678 | - | 6 | 0 |
| Symplocaceae | *Symplocos wikstroemiifolia* | KP094902 | KP093959 | KP092683 | - | 37 | 0 |
| Theaceae | *Camellia euryoides* | KP094740 | KP093803 | #N/A | KP096003 | 29 | 0 |
| Theaceae | *Pyrenaria spectabilis* | KP094934 | KP093989 | KP092647 | - | 13 | 0 |
| Theaceae | *Schima superba* | KP094249 | KP093337 | KP092644 | - | 63 | 12 |
| Iteaceae | *Itea chinensis* | KP094373 | KP093454 | KP092690 | - | 698 | 0 |
| Fabaceae | *Adenanthera pavonina* | KP094938 | KP093993 | KP092694 | - | 0 | 1 |
| Fabaceae | *Archidendron clypearia* | KP094332 | KP093414 | #N/A | KP096006 | 0 | 1 |
| Fabaceae | *Archidendron turgidum* | KP094738 | KP093801 | KP092710 | - | 27 | 3 |
| Fabaceae | *Ormosia semicastrata* | KP094656 | KP093719 | #N/A | KP096015 | 2 | 2 |
| Fabaceae | *Xanthophyllum hainanensis* | KP094662 | KP093725 | KP092743 | - | 4 | 66 |
| Fagaceae | *Castanopsis chinensis* | KP094223 | KP093313 | #N/A | #N/A | 0 | 27 |
| Juglandaceae | *Engelhardia roxburghiana* | KP094930 | KP093986 | KP092757 | - | 3 | 2 |
| Rubiaceae | *Catunaregam spinosa* | KP094947 | KP094000 | KP092782 | - | 0 | 1 |
| Rubiaceae | *Diplospora dubia* | KP094340 | KP093422 | KP092785 | - | 3 | 3 |
| Rubiaceae | *Gardenia jasminoides* | KP094187 | KP093280 | KP092802 | - | 2 | 0 |
| Rubiaceae | *Nauclea officinalis* | KP094282 | KP093368 | KP092795 | - | 0 | 2 |
| Rubiaceae | *Pavetta hongkongensis* | KP094409 | KP093488 | KP092796 | - | 0 | 1 |
| Rubiaceae | *Uncaria rhynchophylla* | KP094819 | KP093879 | KP092803 | - | 16 | 247 |
| Rubiaceae | *Wendlandia uvariifolia* | KP094457 | KP093531 | KP092805 | - | 7 | 1 |
| Lamiaceae | *Clerodendrum cyrtophyllum* | KP094167 | KP093260 | KP092825 | - | 0 | 1 |
| Lamiaceae | *Vitex quinata* | KP094548 | KP093620 | KP092832 | - | 0 | 2 |
| Lauraceae | *Cryptocarya chinensis* | KP094540 | KP093612 | #N/A | KP096041 | 11 | 152 |
| Lauraceae | *Cryptocarya concinna* | KP094293 | KP093379 | KP092863 | - | 8 | 58 |
| Lauraceae | *Lindera chunii* | KP094460 | KP093533 | KP092865 | - | 4 | 10 |
| Lauraceae | *Lindera metcalfiana* | KP094612 | KP093676 | KP092869 | - | 130 | 0 |
| Lauraceae | *Litsea cubeba* | KP094358 | KP093440 | KP092871 | - | 0 | 2 |
| Lauraceae | *Litsea monopetala* | KP094520 | KP093592 | KP092875 | - | 0 | 1 |
| Lauraceae | *Litsea rotundifolia* | KP094181 | KP093274 | KP092876 | - | 16 | 0 |
| Lauraceae | *Machilus breviflora* | KP094423 | KP093502 | KP092878 | - | 178 | 7 |
| Lauraceae | *Machilus chinensis* | KP094530 | KP093602 | KP092879 | - | 2 | 24 |
| Lauraceae | *Machilus kwangtungensis* | KP094532 | KP093604 | KP092881 | - | 363 | 5 |
| Lauraceae | *Machilus phoenicis* | KP094492 | KP093565 | KP092884 | - | 2 | 0 |
| Lauraceae | *Machilus thunbergii* | KP094892 | KP093949 | KP092886 | - | 1 | 0 |
| Lauraceae | *Machilus velutina* | KP094761 | KP093822 | KP092888 | - | 16 | 0 |
| Lauraceae | *Neolitsea cambodiana* | KP094658 | KP093721 | KP092890 | - | 3 | 0 |
| Lauraceae | *Neolitsea umbrosa* | KP094832 | KP093890 | KP092898 | - | 1 | 0 |
| Magnoliaceae | *Magnolia championii* | KP094823 | KP093883 | KP092904 | - | 0 | 44 |
| Clusiaceae | *Garcinia multiflora* | KP095023 | KP094070 | KP092914 | - | 1 | 12 |
| Euphorbiaceae | *Croton lachnocarpus* | KP094558 | KP093630 | KP092920 | - | 1 | 0 |
| Euphorbiaceae | *Mallotus apelta* | KP094219 | KP093309 | KP092931 | - | 0 | 4 |
| Euphorbiaceae | *Mallotus paniculatus* | KP094350 | KP093432 | KP092936 | - | 7 | 26 |
| Hypericaceae | *Cratoxylum cochinchinense* | KP094239 | KP093327 | KP092947 | - | 4 | 0 |
| Phyllanthaceae | *Antidesma japonicum* | KP094443 | KP093522 | KP092952 | - | 0 | 1 |
| Phyllanthaceae | *Aporosa yunnanensis* | KP094680 | KP093743 | KP092958 | - | 0 | 67 |
| Phyllanthaceae | *Bridelia tomentosa* | KP094171 | KP093264 | KP092960 | - | 0 | 1 |
| Phyllanthaceae | *Flueggea virosa* | KP094634 | KP093697 | KP092974 | - | 1 | 2 |
| Phyllanthaceae | *Glochidion eriocarpum* | KP094369 | KP093450 | KP092962 | - | 2 | 0 |
| Phyllanthaceae | *Glochidion puberum* | KP094356 | KP093438 | KP092966 | - | 1 | 0 |
| Salicaceae | *Casearia villilimba* | KP094437 | KP093516 | KP092978 | - | 5 | 1 |
| Malvaceae | *Abutilon indicum* | KP094518 | KP093590 | KP092989 | - | 0 | 30 |
| Malvaceae | *Microcos paniculata* | KP094233 | KP093321 | KP092995 | - | 0 | 10 |
| Malvaceae | *Pterospermum lanceifolium* | KP094676 | KP093739 | KP092997 | - | 0 | 37 |
| Malvaceae | *Sterculia lanceolata* | KP094342 | KP093424 | KP093001 | - | 0 | 42 |
| Thymelaeaceae | *Aquilaria sinensis* | KP094157 | KP093250 | KP093005 | - | 0 | 2 |
| Melastomataceae | *Blastus cochinchinensis* | KP094574 | KP093644 | KP093015 | - | 310 | 50 |
| Melastomataceae | *Melastoma sanguineum* | KP094387 | KP093468 | KP093032 | - | 1 | 0 |
| Melastomataceae | *Memecylon ligustrifolium* | KP094321 | KP093406 | KP093034 | - | 4 | 49 |
| Melastomataceae | *Memecylon nigrescens* | KP094319 | KP093404 | KP093036 | - | 0 | 6 |
| Myrtaceae | *Rhodomyrtus tomentosa* | KP094177 | KP093270 | KP093042 | - | 5 | 0 |
| Myrtaceae | *Syzygium acuminatissimum* | KP094421 | KP093500 | KP093038 | - | 0 | 14 |
| Myrtaceae | *Syzygium hancei* | KP094796 | KP093856 | KP093048 | - | 6 | 0 |
| Myrtaceae | *Syzygium jambos* | KP094169 | KP093262 | KP093050 | - | 6 | 17 |
| Myrtaceae | *Syzygium levinei* | KP094215 | KP093305 | KP093052 | - | 6 | 89 |
| Myrtaceae | *Syzygium rehderianum* | KP094241 | KP093329 | KP093054 | - | 41 | 4 |
| Elaeocarpaceae | *Elaeocarpus chinensis* | KP094451 | KP093525 | KP093060 | poor | 9 | 0 |
| Elaeocarpaceae | *Elaeocarpus japonicus* | KP094874 | KP093931 | KP093061 | - | 10 | 0 |
| Elaeocarpaceae | *Elaeocarpus sylvestris* | KP094623 | KP093686 | KP093064 | - | 7 | 1 |
| Cupressaceae | *Cunninghamia lanceolata* | KP094407 | KP093486 | KP093072 | - | NA | NA |
| Podocarpaceae | *Nageia fleuryi* | KP094315 | KP093400 | KP093069 | - | 0 | 5 |
| Proteaceae | *Helicia reticulata* | KP094684 | KP093747 | KP093078 | - | 72 | 0 |
| Cannabaceae | *Gironniera subaequalis* | KP094668 | KP093731 | KP093083 | - | 0 | 295 |
| Cannabaceae | *Trema tomentosa* | KP095094 | KP094130 | KP093177 | - | 0 | 1 |
| Moraceae | *Artocarpus styracifolius* | KP094328 | KP093412 | KP093087 | - | 4 | 7 |
| Moraceae | *Artocarpus tonkinensis* | KP094622 | KP093685 | KP093089 | - | 0 | 9 |
| Moraceae | *Ficus fistulosa* | KP094399 | KP093478 | #N/A | #N/A | 0 | 2 |
| Moraceae | *Ficus formosana* | KP094538 | KP093610 | KP093099 | - | 61 | 0 |
| Moraceae | *Ficus heterophylla* | KP094636 | KP093699 | KP093102 | - | 0 | 2 |
| Moraceae | *Ficus nervosa* | KP094724 | KP093787 | KP093108 | - | 0 | 13 |
| Moraceae | *Ficus superba* | KP094195 | KP093286 | KP093115 | - | 0 | 1 |
| Moraceae | *Ficus variegata* | KP094199 | KP093290 | KP093116 | - | 0 | 3 |
| Rosaceae | *Rhaphiolepis indica* | KP094360 | KP093442 | KP093147 | - | 2 | 0 |
| Rosaceae | *Rubus rosifolius* | KP095030 | KP094077 | KP093167 | - | 0 | 14 |
| Sabiaceae | *Meliosma rigida* | KP094291 | KP093377 | KP093184 | - | 0 | 4 |
| Schoepfiaceae | *Schoepfia jasminodora* | KP094777 | KP093838 | KP093190 | - | 2 | 0 |
| Anacardiaceae | *Toxicodendron succedaneum* | KP094604 | KP093669 | KP093195 | - | 20 | 0 |
| Burseraceae | *Canarium album* | KP094377 | KP093458 | KP093198 | - | 3 | 26 |
| Rutaceae | *Acronychia pedunculata* | KP094483 | KP093556 | KP093202 | - | 15 | 2 |
| Rutaceae | *Melicope pteleifolia* | KP094243 | KP093331 | KP093207 | - | 4 | 1 |
| Rutaceae | *Zanthoxylum myriacanthum* | KP094536 | KP093608 | KP093219 | - | 6 | 0 |
| Rutaceae | *Zanthoxylum nitidum* | KP094251 | KP093339 | KP093221 | - | 34 | 0 |
| Sapindaceae | *Acer fabri* | KP094586 | KP093653 | #N/A | KP096075 | 0 | 1 |
| Sapindaceae | *Acer tutcheri* | KP094923 | KP093979 | KP093225 | - | 1 | 0 |
| Sapindaceae | *Mischocarpus pentapetalus* | KP094672 | KP093735 | KP093226 | - | 1 | 66 |
| Sapindaceae | *Nephelium chryseum* | KP094303 | KP093389 | KP093228 | - | 0 | 3 |

**Table S2.** General linear mixed effect model (GLMM) results for phylogenetic diversity (PD) as a function of several fixed factors and hierarchical random factors.

| **Model** | ***k*** | **AIC_c_** | **ΔAIC*_c_*** | ***wAIC_c_*** | **R^2^_m_** | **R^2^_c_** |
| --- | --- | --- | --- | --- | --- | --- |
| ~ B + M + R + I | 9 | -21519 | 0 | 0.999 | 9.92 | 72.98 |
| ~ M + R + I | 8 | -21483 | 36 | <0.001 | 9.84 | 72.90 |
| ~ B + M + I | 8 | -20854 | 665 | <0.001 | 8.61 | 71.62 |
| ~ M + I | 7 | -20820 | 699 | <0.001 | 8.54 | 71.55 |
| ~ B + R + I | 8 | -19692 | 1827 | <0.001 | 6.18 | 69.05 |
| ~ R + I | 7 | -19660 | 1859 | <0.001 | 6.10 | 68.98 |
| ~ B + I | 7 | -19628 | 1891 | <0.001 | 6.03 | 68.90 |
| ~ I | 6 | -19596 | 1923 | <0.001 | 5.96 | 68.82 |
| ~ B + M + R | 8 | -19416 | 2103 | <0.001 | 5.57 | 68.41 |
| ~ B + M + R + I2 | 9 | -19415 | 2105 | <0.001 | 5.57 | 68.41 |
| ~ M + R | 7 | -19386 | 2133 | <0.001 | 5.49 | 68.33 |
| ~ M + R + I2 | 8 | -19384 | 2135 | <0.001 | 5.49 | 68.33 |
| ~ B + M + I2 | 8 | -18517 | 3002 | <0.001 | 3.48 | 66.21 |
| ~ B + M | 7 | -18504 | 3015 | <0.001 | 3.45 | 66.17 |
| ~ M + I2 | 7 | -18489 | 3030 | <0.001 | 3.41 | 66.13 |
| ~ M | 6 | -18476 | 3043 | <0.001 | 3.37 | 66.10 |
| ~ B + R + I2 | 8 | -17346 | 4173 | <0.001 | 0.54 | 63.11 |
| ~ R + I2 | 7 | -17320 | 4199 | <0.001 | 0.46 | 63.03 |
| ~ B + R | 7 | -17313 | 4206 | <0.001 | 0.44 | 63.01 |
| ~ R | 6 | -17287 | 4232 | <0.001 | 0.37 | 62.93 |
| ~ B + I2 | 7 | -17222 | 4297 | <0.001 | 0.20 | 62.76 |
| ~ I2 | 6 | -17196 | 4323 | <0.001 | 0.13 | 62.68 |
| ~ B | 6 | -17176 | 4343 | <0.001 | 0.07 | 62.63 |
| ~ 1 | 5 | -17151 | 4369 | <0.001 | 0.00 | 62.55 |

*Notes:* Fixed factors are single plant barcodes (M = *matK*, R = *rbcL*, I = ITS, I2 = ITS2) and family-level backbone (B). Random factors are plots (100 m and 600 m). Values are shown for the estimated number of model parameters (*k*), the information-theoretic Akaike’s information criterion corrected for small samples (AIC_c_), change in AIC_c_ relative to the top-ranked model ($\Delta$AIC_c_), AIC_c_ weight (*w*AIC_c_, model probability), and the marginal and total variance explained (R^2^_m_, R^2^_c_) as a measure of the model’s goodness-of-fit.

**Table S3.** General linear mixed effect model (GLMM) results for mean pairwise distance (MPD) as a function of several fixed factors and hierarchical random factors.

| **Model** | ***k*** | **AIC_c_** | **ΔAIC*_c_*** | ***wAIC_c_*** | **R^2^_m_** | **R^2^_c_** |
| --- | --- | --- | --- | --- | --- | --- |
| ~ B + M + R + I | 9 | -20165 | 0 | 0.999 | 31.34 | 32.18 |
| ~ M + R + I | 8 | -20105 | 60 | <0.001 | 31.04 | 31.88 |
| ~ B + M + R + I2 | 9 | -19618 | 547 | <0.001 | 28.65 | 29.47 |
| ~ M + R + I2 | 8 | -19560 | 604 | <0.001 | 28.35 | 29.17 |
| ~ B + M + R | 8 | -19065 | 1099 | <0.001 | 25.80 | 26.60 |
| ~ M + R | 7 | -19010 | 1155 | <0.001 | 25.50 | 26.30 |
| ~ B + M + I | 8 | -18624 | 1540 | <0.001 | 23.46 | 24.24 |
| ~ M + I | 7 | -18571 | 1594 | <0.001 | 23.16 | 23.94 |
| ~ B + M + I2 | 8 | -18038 | 2127 | <0.001 | 20.22 | 20.99 |
| ~ M + I2 | 7 | -17987 | 2178 | <0.001 | 19.92 | 20.68 |
| ~ B + M | 7 | -17269 | 2896 | <0.001 | 15.75 | 16.48 |
| ~ M | 6 | -17220 | 2945 | <0.001 | 15.45 | 16.18 |
| ~ B + R + I | 8 | -16522 | 3643 | <0.001 | 11.19 | 11.90 |
| ~ R + I | 7 | -16476 | 3689 | <0.001 | 10.89 | 11.60 |
| ~ B + I | 7 | -16348 | 3817 | <0.001 | 10.07 | 10.78 |
| ~ I | 6 | -16303 | 3862 | <0.001 | 9.77 | 10.48 |
| ~ B + R + I2 | 8 | -15968 | 4197 | <0.001 | 7.63 | 8.32 |
| ~ R + I2 | 7 | -15924 | 4241 | <0.001 | 7.33 | 8.02 |
| ~ B + I2 | 7 | -15781 | 4384 | <0.001 | 6.39 | 7.07 |
| ~ I2 | 6 | -15738 | 4427 | <0.001 | 6.09 | 6.77 |
| ~ B + R | 7 | -15148 | 5017 | <0.001 | 2.09 | 2.75 |
| ~ R | 6 | -15107 | 5058 | <0.001 | 1.79 | 2.45 |
| ~ B | 6 | -14895 | 5270 | <0.001 | 0.30 | 0.96 |
| ~ 1 | 5 | -14854 | 5311 | <0.001 | 0.00 | 0.66 |

*Notes:* Fixed factors are single plant barcodes (M = *matK*, R = *rbcL*, I = ITS, I2 = ITS2) and family-level backbone (B). Random factors are plots (100 m and 600 m). Values are shown for the estimated number of model parameters (*k*), the information-theoretic Akaike’s information criterion corrected for small samples (AIC_c_), change in AIC_c_ relative to the top-ranked model ($\Delta$AIC_c_), AIC_c_ weight (*w*AIC_c_, model probability), and the marginal and total variance explained (R^2^_m_, R^2^_c_) as a measure of the model’s goodness-of-fit.

**Table S4.** General linear mixed effect model (GLMM) results for abundance-weighted mean pairwise distance (MPD_ed_) as a function of several fixed factors and hierarchical random factors.

| **Model** | ***k*** | **AIC_c_** | **ΔAIC*_c_*** | ***wAIC_c_*** | **R^2^_m_** | **R^2^_c_** |
| --- | --- | --- | --- | --- | --- | --- |
| ~ B + M + R + I | 9 | -20835 | 0 | 0.999 | 25.03 | 45.34 |
| ~ M + R + I | 8 | -20806 | 29 | <0.001 | 24.91 | 45.22 |
| ~ B + M + R + I2 | 9 | -20103 | 732 | <0.001 | 22.12 | 42.34 |
| ~ M + R + I2 | 8 | -20075 | 760 | <0.001 | 22.00 | 42.21 |
| ~ B + M + I | 8 | -19735 | 1101 | <0.001 | 20.58 | 40.76 |
| ~ M + I | 7 | -19708 | 1127 | <0.001 | 20.46 | 40.63 |
| ~ B + M + R | 8 | -19675 | 1160 | <0.001 | 20.34 | 40.50 |
| ~ M + R | 7 | -19649 | 1186 | <0.001 | 20.22 | 40.37 |
| ~ B + M + I2 | 8 | -18942 | 1893 | <0.001 | 17.16 | 37.22 |
| ~ M + I2 | 7 | -18917 | 1918 | <0.001 | 17.04 | 37.10 |
| ~ B + M | 7 | -18335 | 2500 | <0.001 | 14.38 | 34.36 |
| ~ M | 6 | -18311 | 2524 | <0.001 | 14.26 | 34.24 |
| ~ B + R + I | 8 | -17110 | 3725 | <0.001 | 8.41 | 28.23 |
| ~ R + I | 7 | -17088 | 3747 | <0.001 | 8.28 | 28.10 |
| ~ B + I | 7 | -17067 | 3768 | <0.001 | 8.18 | 27.99 |
| ~ I | 6 | -17046 | 3789 | <0.001 | 8.06 | 27.87 |
| ~ B + R + I2 | 8 | -16364 | 4471 | <0.001 | 4.48 | 24.21 |
| ~ R + I2 | 7 | -16344 | 4491 | <0.001 | 4.36 | 24.08 |
| ~ B + I2 | 7 | -16309 | 4526 | <0.001 | 4.18 | 23.89 |
| ~ I2 | 6 | -16289 | 4546 | <0.001 | 4.05 | 23.77 |
| ~ B + R | 7 | -15678 | 5157 | <0.001 | 0.67 | 20.29 |
| ~ R | 6 | -15659 | 5176 | <0.001 | 0.54 | 20.17 |
| ~ B | 6 | -15585 | 5250 | <0.001 | 0.12 | 19.74 |
| ~ 1 | 5 | -15566 | 5269 | <0.001 | 0.00 | 19.61 |

*Notes:* Fixed factors are single plant barcodes (M = *matK*, R = *rbcL*, I = ITS, I2 = ITS2) and family-level backbone (B). Random factors are plots (100 m and 600 m). Values are shown for the estimated number of model parameters (*k*), the information-theoretic Akaike’s information criterion corrected for small samples (AIC_c_), change in AIC_c_ relative to the top-ranked model ($\Delta$AIC_c_), AIC_c_ weight (*w*AIC_c_, model probability), and the marginal and total variance explained (R^2^_m_, R^2^_c_) as a measure of the model’s goodness-of-fit.

**Table S5.** General linear mixed effect model (GLMM) results for mean nearest-taxon distance (MNTD) as a function of several fixed factors and hierarchical random factors.

| **Model** | ***k*** | **AIC_c_** | **ΔAIC*_c_*** | ***wAIC_c_*** | **R^2^_m_** | **R^2^_c_** |
| --- | --- | --- | --- | --- | --- | --- |
| ~ B + M + R + I | 9 | -6867 | 0 | 0.999 | 11.88 | 78.80 |
| ~ M + R + I | 8 | -6848 | 20 | <0.001 | 11.85 | 78.76 |
| ~ B + M + I | 8 | -4935 | 1933 | <0.001 | 9.09 | 74.69 |
| ~ M + I | 7 | -4918 | 1950 | <0.001 | 9.06 | 74.64 |
| ~ B + M + R + I2 | 9 | -4723 | 2144 | <0.001 | 8.75 | 74.19 |
| ~ M + R + I2 | 8 | -4707 | 2161 | <0.001 | 8.72 | 74.15 |
| ~ B + M + R | 8 | -4571 | 2296 | <0.001 | 8.49 | 73.82 |
| ~ M + R | 7 | -4555 | 2313 | <0.001 | 8.46 | 73.78 |
| ~ B + R + I | 8 | -3714 | 3153 | <0.001 | 6.96 | 71.67 |
| ~ R + I | 7 | -3699 | 3169 | <0.001 | 6.93 | 71.62 |
| ~ B + I | 7 | -3296 | 3571 | <0.001 | 6.15 | 70.55 |
| ~ I | 6 | -3282 | 3586 | <0.001 | 6.12 | 70.50 |
| ~ B + M + I2 | 8 | -2680 | 4188 | <0.001 | 4.89 | 68.82 |
| ~ M + I2 | 7 | -2666 | 4202 | <0.001 | 4.86 | 68.77 |
| ~ B + M | 7 | -2366 | 4501 | <0.001 | 4.21 | 67.89 |
| ~ M | 6 | -2353 | 4515 | <0.001 | 4.17 | 67.85 |
| ~ B + R + I2 | 8 | -1537 | 5331 | <0.001 | 2.29 | 65.33 |
| ~ R + I2 | 7 | -1524 | 5344 | <0.001 | 2.26 | 65.28 |
| ~ B + R | 7 | -1190 | 5677 | <0.001 | 1.43 | 64.18 |
| ~ R | 6 | -1178 | 5689 | <0.001 | 1.40 | 64.14 |
| ~ B + I2 | 7 | -1070 | 5797 | <0.001 | 1.13 | 63.78 |
| ~ I2 | 6 | -1058 | 5810 | <0.001 | 1.09 | 63.73 |
| ~ B | 6 | -658 | 6210 | <0.001 | 0.04 | 62.35 |
| ~ 1 | 5 | -646 | 6222 | <0.001 | 0.00 | 62.31 |

*Notes:* Fixed factors are single plant barcodes (M = *matK*, R = *rbcL*, I = ITS, I2 = ITS2) and family-level backbone (B). Random factors are plots (100 m and 600 m). Values are shown for the estimated number of model parameters (*k*), the information-theoretic Akaike’s information criterion corrected for small samples (AIC_c_), change in AIC_c_ relative to the top-ranked model ($\Delta$AIC_c_), AIC_c_ weight (*w*AIC_c_, model probability), and the marginal and total variance explained (R^2^_m_, R^2^_c_) as a measure of the model’s goodness-of-fit.

**Table S6.** General linear mixed effect model (GLMM) results for abundance-weighted mean nearest-taxon distance (MNTD_ed_) as a function of several fixed factors and hierarchical random factors.

| **Model** | ***k*** | **AIC_c_** | **ΔAIC*_c_*** | ***wAIC_c_*** | **R^2^_m_** | **R^2^_c_** |
| --- | --- | --- | --- | --- | --- | --- |
| ~ B + M + R + I | 9 | -11181 | 0 | 0.999 | 15.11 | 63.78 |
| ~ M + R + I | 8 | -11150 | 31 | <0.001 | 15.03 | 63.69 |
| ~ B + M + R + I2 | 9 | -10583 | 598 | <0.001 | 13.52 | 62.25 |
| ~ M + R + I2 | 8 | -10553 | 627 | <0.001 | 13.43 | 62.17 |
| ~ B + M + R | 8 | -10031 | 1150 | <0.001 | 11.97 | 60.78 |
| ~ M + R | 7 | -10003 | 1178 | <0.001 | 11.87 | 60.75 |
| ~ B + M + I | 8 | -9630 | 1551 | <0.001 | 10.82 | 59.69 |
| ~ M + I | 7 | -9603 | 1578 | <0.001 | 10.74 | 59.60 |
| ~ B + M + I2 | 8 | -8991 | 2190 | <0.001 | 8.92 | 57.88 |
| ~ M + I2 | 7 | -8965 | 2216 | <0.001 | 8.84 | 57.79 |
| ~ B + M | 7 | -8221 | 2959 | <0.001 | 6.52 | 55.57 |
| ~ M | 6 | -8197 | 2984 | <0.001 | 6.44 | 55.50 |
| ~ B + R + I | 8 | -8098 | 3082 | <0.001 | 6.14 | 55.21 |
| ~ R + I | 7 | -8074 | 3107 | <0.001 | 6.05 | 55.12 |
| ~ B + I | 7 | -7845 | 3336 | <0.001 | 5.31 | 54.42 |
| ~ I | 6 | -7821 | 3360 | <0.001 | 5.22 | 54.33 |
| ~ B + R + I2 | 8 | -7483 | 3697 | <0.001 | 4.12 | 53.26 |
| ~ R + I2 | 7 | -7460 | 3721 | <0.001 | 4.03 | 53.18 |
| ~ B + I2 | 7 | -7215 | 3966 | <0.001 | 3.20 | 52.40 |
| ~ I2 | 6 | -7192 | 3988 | <0.001 | 3.12 | 52.31 |
| ~ B + R | 7 | -6672 | 4509 | <0.001 | 1.32 | 50.58 |
| ~ R | 6 | -6650 | 4531 | <0.001 | 1.23 | 50.51 |
| ~ B | 6 | -6328 | 4852 | <0.001 | 0.08 | 49.39 |
| ~ 1 | 5 | -6307 | 4874 | <0.001 | 0.00 | 49.32 |

*Notes:* Fixed factors are single plant barcodes (M = *matK*, R = *rbcL*, I = ITS, I2 = ITS2) and family-level backbone (B). Random factors are plots (100 m and 600 m). Values are shown for the estimated number of model parameters (*k*), the information-theoretic Akaike’s information criterion corrected for small samples (AIC_c_), change in AIC_c_ relative to the top-ranked model ($\Delta$AIC_c_), AIC_c_ weight (*w*AIC_c_, model probability), and the marginal and total variance explained (R^2^_m_, R^2^_c_) as a measure of the model’s goodness-of-fit.

**Table S7.** General linear mixed effect model (GLMM) results for phylogenetic abundance evenness (PAE) as a function of several fixed factors and hierarchical random factors.

| **Model** | ***k*** | **AIC_c_** | **ΔAIC*_c_*** | ***wAIC_c_*** | **R^2^_m_** | **R^2^_c_** |
| --- | --- | --- | --- | --- | --- | --- |
| ~ B + M + R + I | 9 | -35388 | 0 | 0.999 | 21.14 | 46.87 |
| ~ B + R + I | 8 | -34775 | 613 | <0.001 | 18.75 | 44.48 |
| ~ M + R + I | 8 | -34729 | 660 | <0.001 | 18.57 | 44.29 |
| ~ B + M + I | 8 | -34538 | 850 | <0.001 | 17.81 | 43.53 |
| ~ B + I | 7 | -34354 | 1035 | <0.001 | 17.05 | 42.77 |
| ~ R + I | 7 | -34144 | 1245 | <0.001 | 16.18 | 41.91 |
| ~ M + I | 7 | -33917 | 1471 | <0.001 | 15.24 | 40.96 |
| ~ I | 6 | -33741 | 1648 | <0.001 | 14.48 | 40.20 |
| ~ B + M + R + I2 | 9 | -32944 | 2444 | <0.001 | 11.01 | 36.72 |
| ~ B + M + R | 8 | -32609 | 2779 | <0.001 | 9.46 | 35.18 |
| ~ M + R + I2 | 8 | -32389 | 3000 | <0.001 | 8.43 | 34.15 |
| ~ M + R | 7 | -32066 | 3322 | <0.001 | 6.89 | 32.61 |
| ~ B + R + I2 | 8 | -31917 | 3471 | <0.001 | 6.18 | 31.91 |
| ~ B + R | 7 | -31737 | 3651 | <0.001 | 5.29 | 31.00 |
| ~ B + M + I2 | 8 | -31649 | 3739 | <0.001 | 4.86 | 30.57 |
| ~ B + M | 7 | -31487 | 3901 | <0.001 | 4.04 | 29.76 |
| ~ R + I2 | 7 | -31400 | 3988 | <0.001 | 3.61 | 29.32 |
| ~ B + I2 | 7 | -31319 | 4070 | <0.001 | 3.19 | 28.91 |
| ~ R | 6 | -31227 | 4162 | <0.001 | 2.72 | 28.43 |
| ~ B | 6 | -31199 | 4190 | <0.001 | 2.57 | 28.28 |
| ~ M + I2 | 7 | -31142 | 4247 | <0.001 | 2.29 | 28.00 |
| ~ M | 6 | -30986 | 4403 | <0.001 | 1.47 | 27.18 |
| ~ I2 | 6 | -30823 | 4565 | <0.001 | 0.62 | 26.33 |
| ~ 1 | 5 | -30707 | 4681 | <0.001 | 0.00 | 25.71 |

*Notes:* Fixed factors are single plant barcodes (M = *matK*, R = *rbcL*, I = ITS, I2 = ITS2) and family-level backbone (B). Random factors are plots (100 m and 600 m). Values are shown for the estimated number of model parameters (*k*), the information-theoretic Akaike’s information criterion corrected for small samples (AIC_c_), change in AIC_c_ relative to the top-ranked model ($\Delta$AIC_c_), AIC_c_ weight (*w*AIC_c_, model probability), and the marginal and total variance explained (R^2^_m_, R^2^_c_) as a measure of the model’s goodness-of-fit.

**Table S8.** General linear mixed effect model (GLMM) results for imbalance of abundance among clades (IAC) as a function of several fixed factors and hierarchical random factors.

| **Model** | ***k*** | **AIC_c_** | **ΔAIC*_c_*** | ***wAIC_c_*** | **R^2^_m_** | **R^2^_c_** |
| --- | --- | --- | --- | --- | --- | --- |
| ~ B + M + R + I2 | 9 | -53553 | 0 | 0.999 | 0.034 | 99.893 |
| ~ B + M + R + I | 9 | -53460 | 94 | <0.001 | 0.033 | 99.892 |
| ~ B + M + R | 8 | -53434 | 120 | <0.001 | 0.033 | 99.892 |
| ~ B + R + I2 | 8 | -53251 | 302 | <0.001 | 0.032 | 99.890 |
| ~ B + R + I | 8 | -53076 | 477 | <0.001 | 0.030 | 99.888 |
| ~ B + R | 7 | -53072 | 482 | <0.001 | 0.030 | 99.888 |
| ~ B + M + I2 | 8 | -52621 | 933 | <0.001 | 0.027 | 99.884 |
| ~ B + I2 | 7 | -52597 | 956 | <0.001 | 0.027 | 99.884 |
| ~ B + M | 7 | -52398 | 1156 | <0.001 | 0.025 | 99.882 |
| ~ B + M + I | 8 | -52396 | 1157 | <0.001 | 0.025 | 99.882 |
| ~ B | 6 | -52359 | 1194 | <0.001 | 0.025 | 99.882 |
| ~ B + I | 7 | -52357 | 1196 | <0.001 | 0.025 | 99.882 |
| ~ M + R + I2 | 8 | -50681 | 2872 | <0.001 | 0.010 | 99.864 |
| ~ M + R + I | 8 | -50605 | 2949 | <0.001 | 0.009 | 99.863 |
| ~ M + R | 7 | -50584 | 2969 | <0.001 | 0.009 | 99.863 |
| ~ R + I2 | 7 | -50435 | 3119 | <0.001 | 0.007 | 99.862 |
| ~ R + I | 7 | -50292 | 3262 | <0.001 | 0.006 | 99.860 |
| ~ R | 6 | -50289 | 3265 | <0.001 | 0.006 | 99.860 |
| ~ M + I2 | 7 | -49917 | 3636 | <0.001 | 0.002 | 99.855 |
| ~ I2 | 6 | -49898 | 3655 | <0.001 | 0.002 | 99.855 |
| ~ M | 6 | -49734 | 3820 | <0.001 | 0.000 | 99.853 |
| ~ M + I | 7 | -49732 | 3821 | <0.001 | 0.000 | 99.853 |
| ~ 1 | 5 | -49702 | 3851 | <0.001 | 0.000 | 99.853 |
| ~ I | 6 | -49700 | 3853 | <0.001 | 0.000 | 99.853 |

*Notes:* Fixed factors are single plant barcodes (M = *matK*, R = *rbcL*, I = ITS, I2 = ITS2) and family-level backbone (B). Random factors are plots (100 m and 600 m). Values are shown for the estimated number of model parameters (*k*), the information-theoretic Akaike’s information criterion corrected for small samples (AIC_c_), change in AIC_c_ relative to the top-ranked model ($\Delta$AIC_c_), AIC_c_ weight (*w*AIC_c_, model probability), and the marginal and total variance explained (R^2^_m_, R^2^_c_) as a measure of the model’s goodness-of-fit.

**Figure S1.** Bayesian phylogenetic trees based on *matK* + ITS2 barcodes under enforcement of a backbone phylogeny using family relationships from APG IV (a) or not (b) with node values indicating estimated posterior probabilities from Bayesian analysis.


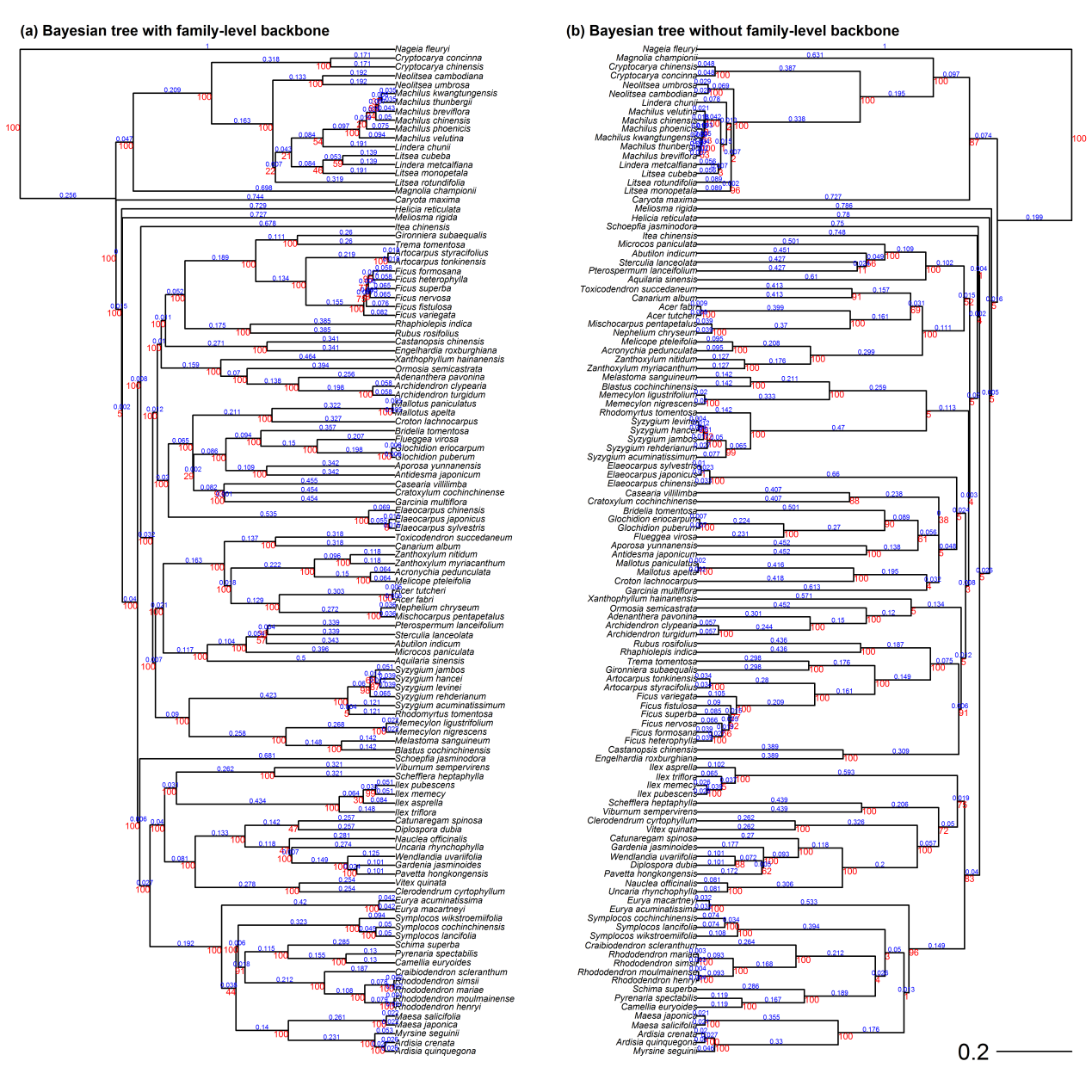


**Figure S2.** Bayesian phylogenetic trees based on *matK* + ITS barcodes under enforcement of a backbone phylogeny using family relationships from APG IV (a) or not (b) with node values indicating estimated posterior probabilities from Bayesian analysis.


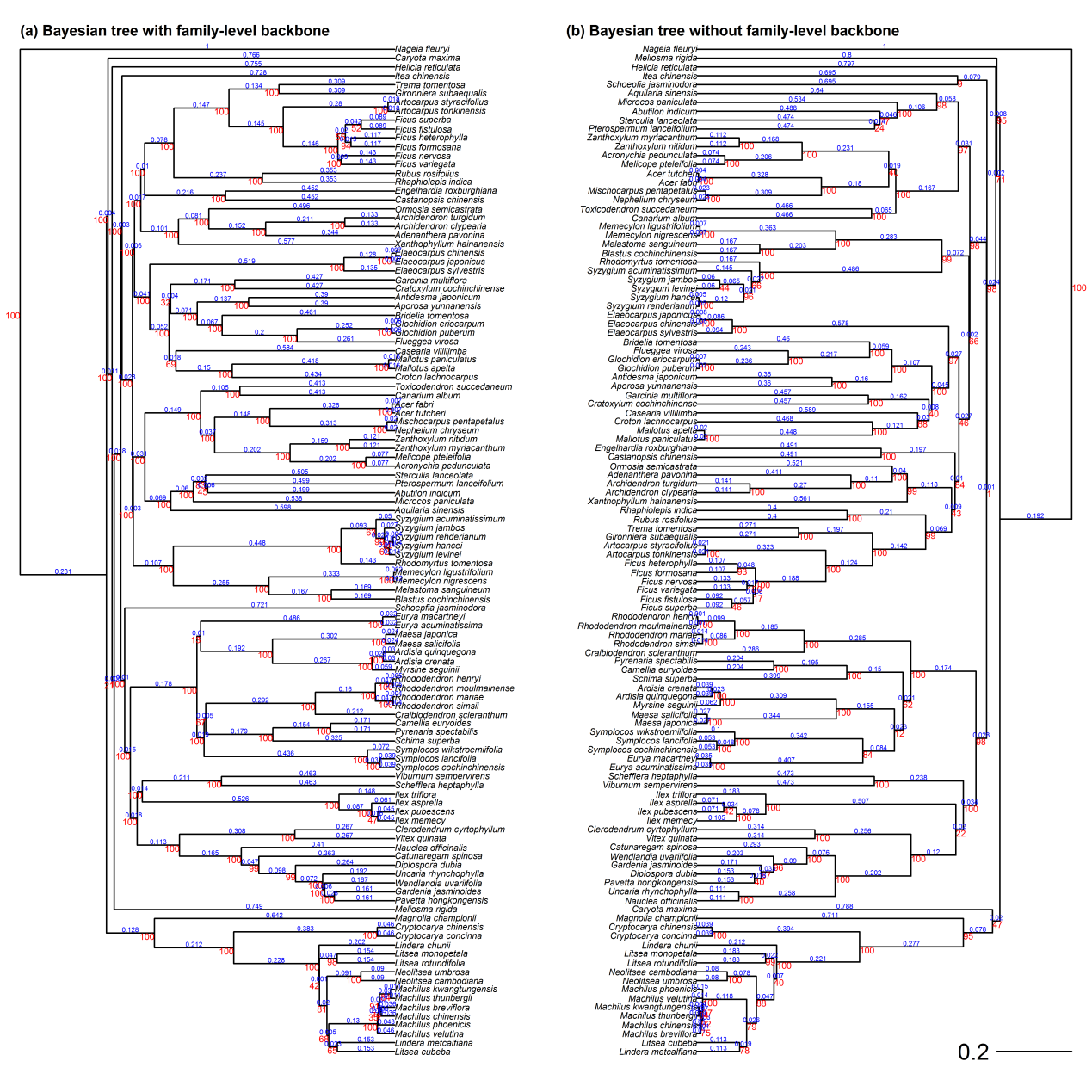


**Figure S3.** Bayesian phylogenetic trees based on *rbcL* + ITS2 barcodes under enforcement of a backbone phylogeny using family relationships from APG IV (a) or not (b) with node values indicating estimated posterior probabilities from Bayesian analysis.


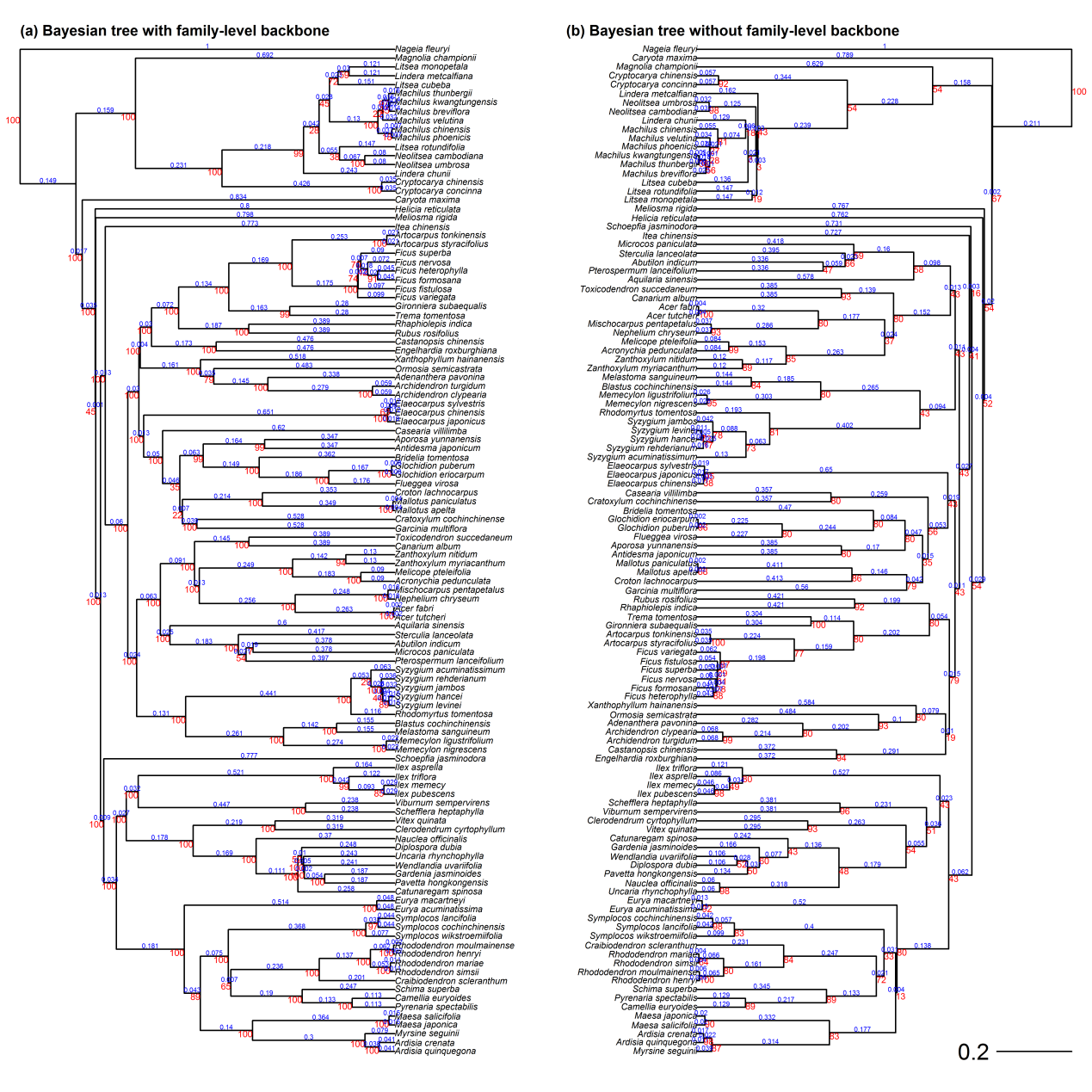


**Figure S4.** Bayesian phylogenetic trees based on *rbcL* + ITS barcodes under enforcement of a backbone phylogeny using family relationships from APG IV (a) or not (b) with node values indicating estimated posterior probabilities from Bayesian analysis.


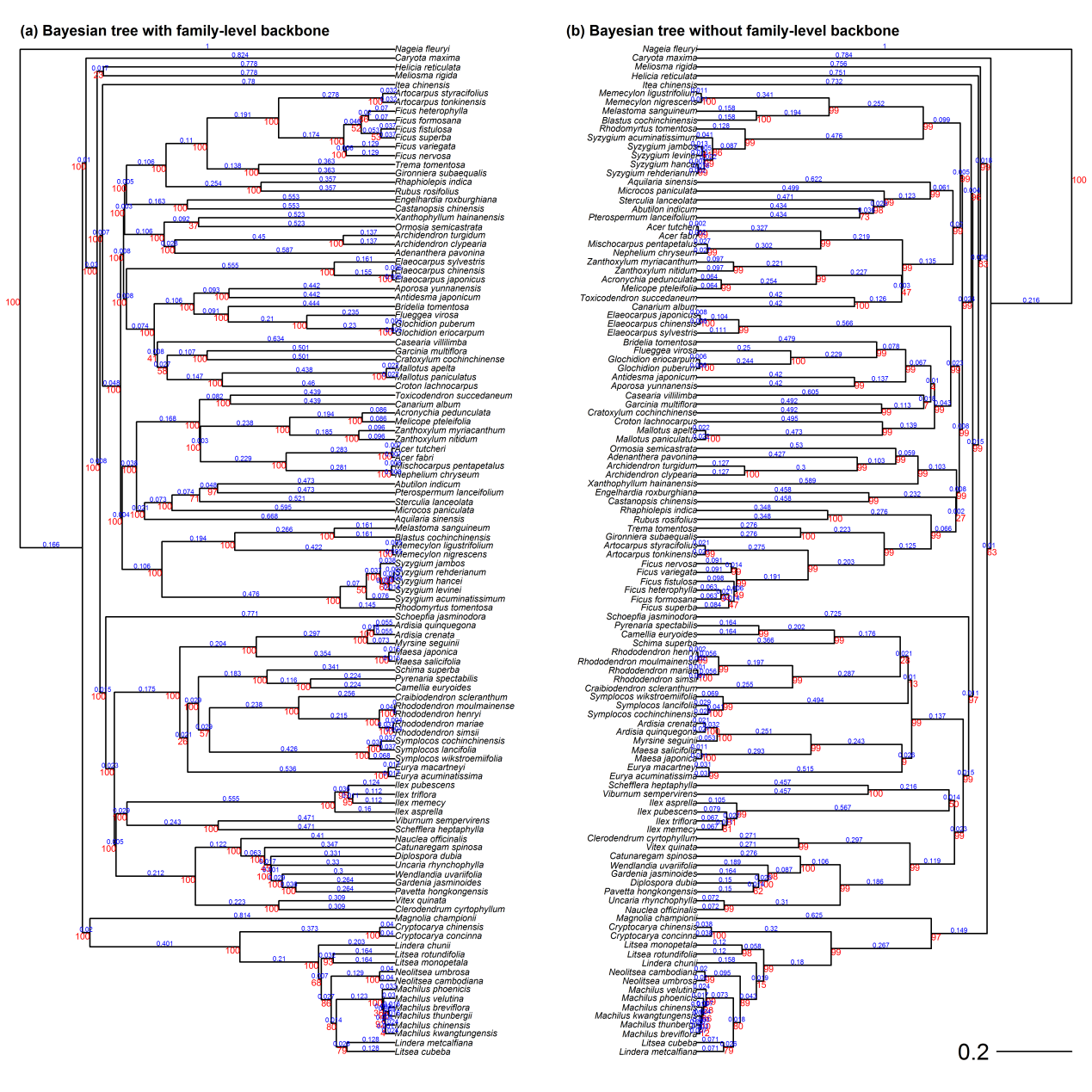


**Figure S5.** Bayesian phylogenetic trees based on *rbcL* + *matK* barcodes under enforcement of a backbone phylogeny using family relationships from APG IV (a) or not (b) with node values indicating estimated posterior probabilities from Bayesian analysis.


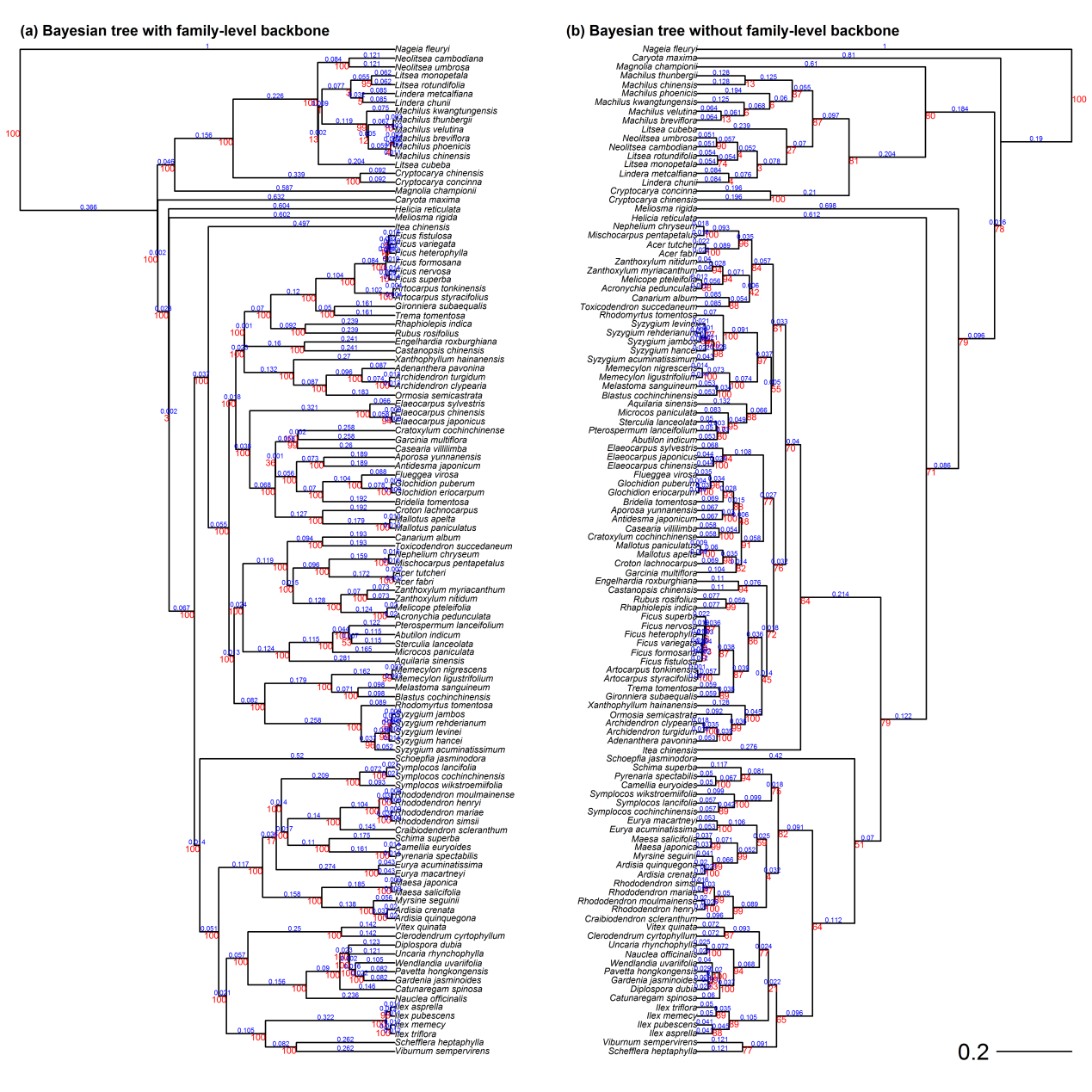


**Figure S6.** Bayesian phylogenetic trees based on *rbcL* + *matK* + ITS2 barcodes under enforcement of a backbone phylogeny using family relationships from APG IV (a) or not (b) with node values indicating estimated posterior probabilities from Bayesian analysis.


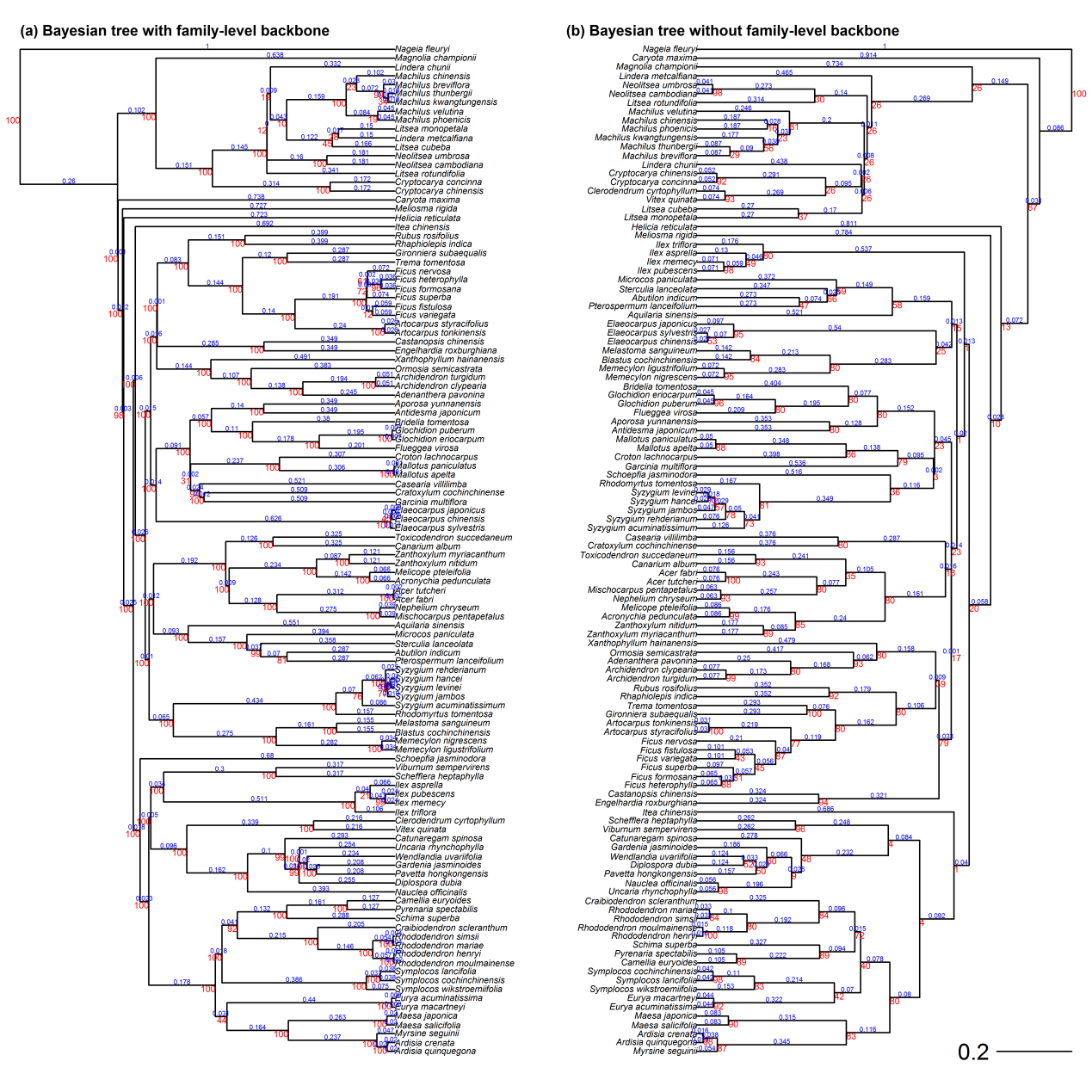


**Figure S7.** Bayesian phylogenetic trees based on *rbcL* + *matK* + ITS barcodes under enforcement of a backbone phylogeny using family relationships from APG IV (a) or not (b) with node values indicating estimated posterior probabilities from Bayesian analysis.


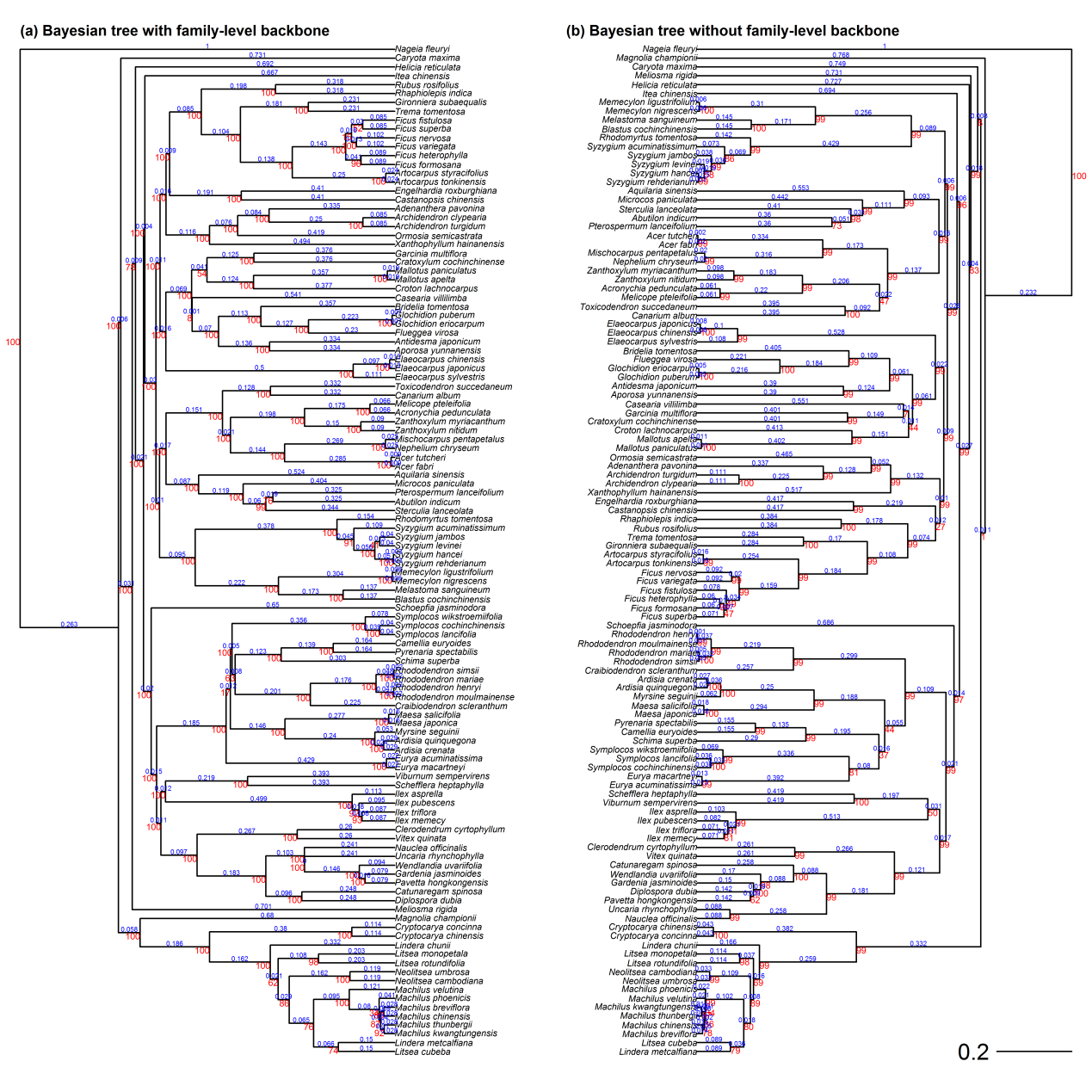

Supplement: Supplementary file 1 [file ECE3-9-5372-s001.docx]
